# Strong innate immune response and cell death in chicken splenocytes infected with genotype VIId Newcastle disease virus

**DOI:** 10.1186/1743-422X-9-208

**Published:** 2012-09-18

**Authors:** Zenglei Hu, Jiao Hu, Shunlin Hu, Xiaowen Liu, Xiaoquan Wang, Jie Zhu, Xiufan Liu

**Affiliations:** 1 Animal Infectious Disease Laboratory, School of Veterinary Medicine, Yangzhou University, 12 East Wenhui Road, Yangzhou Jiangsu Province, 225009, China; 2 Ministry of Education Key Lab for Avian Preventive Medicine, School of Veterinary Medicine, Yangzhou University, 12 East Wenhui Road, Yangzhou Jiangsu Province, 225009, China

**Keywords:** Innate immune response, Cell death, Splenic necrosis, Genotype VIId Newcastle disease virus

## Abstract

**Background:**

Genotype VIId Newcastle disease virus (NDV) isolates induce more severe damage to lymphoid tissues, especially to the spleen, when compared to virulent viruses of other genotypes. However, the biological basis of the unusual pathological changes remains largely unknown.

**Methods:**

Virus replication, cytokine gene expression profile and cell death response in chicken splenocytes infected with two genotype VIId NDV strains (JS5/05 and JS3/05), genotype IX NDV strain F48E8 and genotype IV NDV strain Herts/33 were evaluated. Statistical significance of differences between experimental groups was determined using the Independent-Samples T test.

**Results:**

JS5/05 and JS3/05 caused hyperinduction of type I interferons (IFNs) (IFN-α and -β) during detection period compared to F48E8 and Herts/33. JS5/05 increased expression level of IFN-γ gene at 6 h post-inoculation (pi) and JS3/05 initiated sustained activation of IFN-γ within 24 h pi, whereas transcriptional levels of IFN-γ remained unchanged at any of the time points during infection of F48E8 and Herts/33. In addition, compared to F48E8 and Herts/33, JS3/05 and JS5/05 significantly increased the amount of free nucleosomal DNA in splenocytes at 6 and 24 h pi respectively. Annexin-V and Proidium iodid (PI) double staining of infected cells showed that cell death induced by JS3/05 and JS5/05 was characterized by marked necrosis compared to F48E8 and Herts/33 at 24 h pi. These results indicate that genotype VIId NDV strains JS3/05 and JS5/05 elicited stronger innate immune and cell death responses in chicken splenocytes than F48E8 and Herts/33. JS5/05 replicated at a significantly higher efficiency in splenocytes than F48E8 and Herts/33. Early excessive cell death induced by JS3/05 infection partially impaired virus replication.

**Conclusions:**

Viral dysregulaiton of host response may be relevant to the severe pathological manifestation in the spleen following genotype VIId NDV infection.

## Background

Genotype VIId Newcastle disease virus (NDV) is dominant in Asia [1-3]. Many pathological studies have revealed that genotype VIId NDV isolates induce the most severe necrosis in lymphoid tissues, especially in the spleen, when compared with virulent isolates of other genotypes [4-6]. However, the basis for the unusual pathological manifestation remains unclear.

Although many viral determinants for pathogenicity of NDV have been identified [7-9], few mechanistic data regarding host response to NDV infection are available. Some recent studies have demonstrated that viral modulation of host innate immune response is associated with viral pathogenesis and pathological outcomes [4,10]. Similarly, numerous studies in animal and cell models have shown that robust host immune response contributes to the pathogenesis of highly pathogenic avian H5N1 influenza virus [11,12].

In addition, apoptosis is associated with the pathogenesis of NDV. Chickens experimentally infected with virulent NDV isolates exhibited prominent apoptosis in lymphoid tissues [13-15]. Moreover, Harrison et al. have shown that the degree of apoptosis in lymphoid tissues is correlated with the severity of disease caused by NDV strains of varying virulence [15].

Based on these findings, we hypothesize that host innate immune response and apoptosis are associated with the severe destruction of lymphoid tissues following genotype VIId NDV infection.

Considering that results in primary cultured cells can be closely correlated with pathogenesis *in vivo*, we used chicken splenocytes as an *in vitro* model to compare apoptosis and cytokine response following infection with two genotype VIId NDV strains JS5/05 and JS3/05, a genotype IX NDV strain F48E8 and a genotype IV NDV strain Herts/33.

## Results

### Virus replication in splenocytes

**Figure 1:**
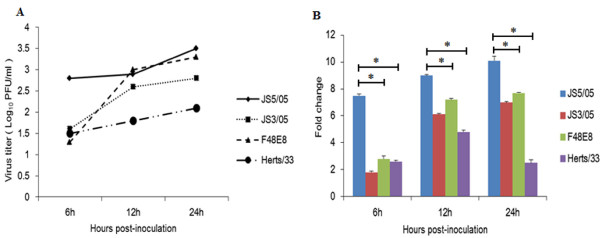
** Characterization of virus replication in splenocytes.** (**A**) Virus titration of culture supernatants from virus-infected splenocytes using plaque formation test in CEF. (**B**) Analysis of viral M gene transcription profiles using qRT-PCR. The transcription levels of viral M gene were normalized to those of β-actin gene in the corresponding sample. Asterisk (*) indicates significant difference at *p* < 0.05 (n = 3) between experimental groups.

**Figure 2:**
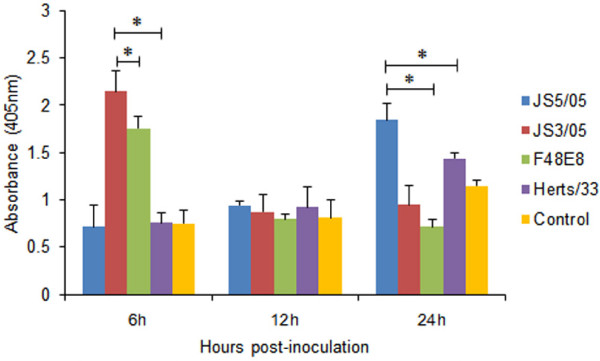
** Detection of free nucleosomal DNA in the cytoplasm of infected splenocytes.** Cytoplasmic fractions of inoculated cells were used for detection of fragmented DNA by ELISA. Asterisk (*) indicates significant difference at *p* < 0.05 (n = 3) between experimental groups.

To evaluate difference in replication of NDV strains from different genotypes in chicken splenocytes, we determined virus amounts in culture supernatants and transcriptional levels of viral matrix (M) gene. Titration test showed that culture supernatants of JS5/05-inoculated splenocytes had the largest amount of virus at 6 h post-inoculation (pi) than those of cells inoculated with other tested viruses (Figure 1A). In addition, JS5/05 maintained this high replication efficiency during detection period. At 12 and 24 h pi, F48E8 also showed high replication efficiency in splenocytes, which is consistent with its high virus titer in chicken embryo fibroblasts (CEF) (Table 1). Moreover, JS3/05 replicated with relative low efficiency when compared to JS5/05 and F48E8. Splenocytes inoculated with Herts/33 released the lowest amount of virus into culture supernatants among these four NDV strains. Data of quantitative real-time polymerase chain reaction (qRT-PCR) agreed with the results of titration assay (Figure 1B). M gene transcription levels of JS5/05 strain were significantly higher than those of F48E8 and Herts/33 strain at any of the time points. However, JS3/05 showed relative low M gene expression levels than JS5/05 and F48E8, which may be attributed to the early cell death induced by JS3/05 infection (Figure 2). These results suggest that high replication efficiency of genotype VIId NDV in splenocytes was isolate-specific and NDV replication may be partially associated with virus-induced cell death.

**Table 1:** Background information of NDV strains used in this study

**Virus**	**Host**	**Year of isolation**	**Genotype**	**Accession number**	**Titer (TCID**_**50**_**/ml)**
JS5/05	Goose	2005	VIId	JN631747	10^9.2^
JS3/05	Fowl	2005	VIId	JN618349	10^9.2^
F48E8	Fowl	1946	IX	AY260113	10^10.3^
Herts/33	Fowl	1933	IV	AY741404	10^8.6^

### Apoptosis assay

We next quantitatively determined cytoplasmic histone-associated-DNA-fragments using enzyme linked immunosorbent assay (ELISA) to evaluate virus-induced apoptosis. We only focused on the difference in apoptosis between genotype VIId NDV strains and viruses of other genotypes. As shown in Figure 2, at 6 h pi, the absorbance value of splenocytes inoculated with JS3/05 was significantly higher than that of cells inoculated with F48E8 and Herts/33. At 12 h pi, the absorbance values of splenocytes infected with all viruses were comparable. At 24 h pi, JS5/05-inocualted cells produced significantly higher absorbance values than those of F48E8- and Herts-inoculated cells. These findings demonstrated that splenocytes infected with genotype VIId NDVs (JS5/05 and JS3/05) were richer in free nucleosomal DNA than those infected with F48E8 and Herts/33, indicating that JS3/05 and JS5/05 induced stronger apoptosis in splenocytes when compared to F48E8 and Herts/33.

### Cytokine gene expression profile

**Figure 3:**
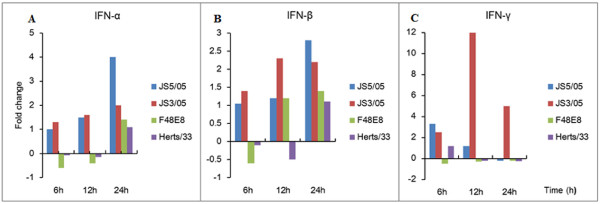
** Determination of mRNA expression of IFN-α, IFN-β and IFN-γ genes in the splenocytes.** Profiles of IFN-α, IFN-β and IFN-γ gene expression were shown in panel **A**, **B** and **C** respectively. The standard curve method was used to analyze the fold change of relative gene expression levels between mock-infected and infected cells. Relative expression levels were normalized to the internal β-actin. The data are the mean fold change ± standard deviation (SD).

Interferons (IFNs) are important members of host innate arm of immunity to prevent virus infection. However, overproduction of IFNs is also related to cytokine storm that contributes to viral immunopathogenesis. We used qRT-PCR to characterize expression profiles of IFN genes, including IFN-α, IFN-β and IFN-γ, to provide insights into the role of innate immune response in pathogenesis of NDV. The results showed that JS3/05 and JS5/05 slightly upregulated expression level of IFN-α at 6 and 12 h pi and these two viruses increased expression values of IFN-α at 24 h pi (4-fold for JS5/05 and 2-fold for JS3/05) (Figure 3A). However, in F48E8- or Herts/33-infected cells, the transcription levels of IFN-α did not appear to change at any of the time points (Figure 3A). Transcription levels of IFN-β did not show notable changes at 6 h pi in cells infected with four tested NDVs. At 12 h pi, only JS3/05-inoculated cells showed a 2.3-fold increase of IFN-β expression. At 24 h pi, a 2.8-fold increase of IFN-β expression was detected in splenocytes infected with JS5/05 and 2.2-fold increase for JS3/05. In contrast, IFN-β remained unchanged during detection period in F48E8- and Herts/33-inoculated cells (Figure 3B). IFN-γ RNA amounts were increased by two genotype VIId NDV strains (3.3-fold for JS5/05 and 2.5-fold for JS3/05) at 6 h pi. IFN-γ expression levels in JS5/05-inocualted cells decreased at a stepwise pattern at 12 and 24 h pi. JS3/05 increased expression level of IFN-γ gene up to 12-fold at 12 h pi and high value (5-fold increase) was also observed at 24 h pi. However, no marked change in IFN-γ expression was found for F48E8 and Herts/33 (Figure 3C). These results suggest that JS5/05 and JS3/05 triggered a more potent innate immune response compared to F48E8 and Herts/33.

### Annexin-V and PI double staining

**Figure 4:**
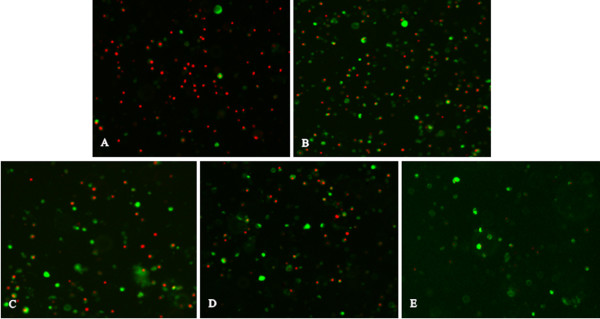
** Annexin-V and PI doubling staining of virus-inoculated splenocytes.** (**A**) JS3/05. (**B**) JS5/05. (**C**) F48E8. (**D**) Herts/33. (**E**) Control. Green: early apoptotic cells with PS exposure upon the outer leaflet of the cell membrane (Annexin-V +); Green and red: late necrotic cells with PS exposure and the ruptured membrane (Annexin-V/PI +); Red: released cellular DNA from leaky necrotic cells (PI +). Magnification, × 400.

Virus-induced cytopathic effect (CPE) is related to virus replication efficiency in host cells and virus pathogenicity. Apoptosis is a critical mechanism of NDV-induced CPE. We then used Annexin-V and PI double staining that can differentiate early apoptotic cells and necrotic cells to evaluate morphological changes caused by NDV isolates of different genotypes. At 24 h pi, splenocyets infected with JS3/05 were characterized with the presence of large amount of cellular DNA released from lysed necrotic cells. Many apoptotic and necrotic cells coexisted in splenocytes infected with JS5/05, and these necrotic cells did not completely lose membrane integrity (Figure 4). This finding is consistent with the high level of free nucleosomal DNA detected using ELISA in JS5/05-inoculated cells at 24 h pi (Figure 2). In contrast, there were fewer apoptotic and necrotic cells in splenocytes infected with F48E8 and Herts/33 (Figure 4). These results showed that in addition to apoptosis, genotype VIId NDV isolates also induced marked cellular necrosis compared to F48E8 and Herts/33, which may have effect on virus replication and viral pathogenicity.

## Discussion

The biological basis of severe tissue damage in the lymphoid system induced by genotype VIId NDV strains is unknown. We hypothesized that dysregulation of host response may play an important role. In this study, our findings demonstrated that two isolates (JS5/05 and JS3/05) of genotype VIId NDV elicited robust innate immune response that was evidenced by upregualtion of IFNs expression compared to genotype IX NDV F48E8 and genotype IV NDV Herts/33. In addition, JS5/05 and JS3/05 also induced enhanced apoptosis and necrosis compared to F48E8 and Herts/33. Virus replication was observed with partial association with virus-induced cell death. Therefore, our *in vitro* results indicate that dysregulation of host response may contribute to the severe spleen pathology caused by genotype VIId NDV infection.

Host cells mount the cytokine response to prevent infection upon virus invasion. However, overproduction of cytokines may form a cytokine storm to amplify detrimental effect of inflammation on the host [16]. In this study, quantitative analysis of transcription levels of IFN genes showed that JS5/05 and JS3/05 increased expression levels of IFN-α, IFN-β and IFN-γ compared to F48E8 and Herts/33, indicating that genotype VIId NDV strains induce a more potent IFN response (Figure 3). Therefore, strong activation of IFN response may intensify inflammation that causes damages in tissue structures. It is notable that hyperinduction of IFN-γ, a key pro-inflammatory cytokine, may increase synthesis of nitric oxide that has toxic effect on host. IFNs are a family of cytokines that play a central role in innate immunity to viruses. The correlation of JS5/05-induced high levels of IFNs and high virus load may result from the faster virus replication to overcome anti-viral effect of IFNs. Additionally, NDV V protein serves as an antagonist of alpha IFN and contributes to viral virulence [17]. Our data showed that at least at mRNA level, a block of IFN function did not occur during infection with JS5/05 and JS3/05. Perhaps these two isolates can activate many genes that involved in IFN pathway to overwhelm the suppression effect of V protein.

In addition to the role in inflammation and anti-virus activity, IFNs also act as key mediators of apoptosis [18]. Many factors involved in IFN signaling pathway, such as IFN regulatory factors (IRFs), protein kinase R (PKR) and 2',5-oligoadenylate/RNaseL system, contribute to apoptosis through different mechanisms. IFNs can also induce the activation of TNF-related apoptosis-inducing ligand (TRAIL) that initiates the death receptor-mediated cell death [18].

Here we showed that JS5/05 and JS3/05 induced stronger apoptosis than F48E8 and Herts/33, reflected by the significantly larger amount of free nucleosomal DNA in JS5/05- and JS3/05-inucolated cells (Figure 2). In addition, splenocytes inoculated with JS3/05 and JS5/05 also underwent marked necrosis (Figure 4). One possible consequence is that stronger cell death in splenocytes infected with JS5/05 and JS3/05 may accelerate disease severity. It has been shown that apoptosis in lymphoid tissues in NDV-infected birds was followed by extensive lymphocellular necrosis [13,14]. Harrison et al. have demonstrated that the apoptotic rate is proportional to the disease severity produced by NDV strains of varying virulence [15]. Accumulating evidence has shown that the pathogenicity of avian influenza virus H5N1 is closely associated with virus-induced apoptosis [19-21]. Of particular interest, as an alternate form of programmed cell death, necrosis may have different effects on disease severity from that of apoptosis. Necrotic cells will release the cytoplasmic contents into the extracellular fluid to stimulate inflammatory response [22].

Based on our findings, we could speculate that cytokine response and cell death may act in concert to exacerbate the disease severity caused by genotype VIId NDV. Our results also indicate that viral dysregulation of host response by genotype VIId NDV strains may be a genotype-specific phenotype.

It is commonly assumed that efficiency of virus replication is an important determinant for the severity of disease. NDV can propagate in chicken lymphocytes and cause lymphocyte depletion, which lead to severe consequences for bird immune system [13,14,23,24]. Replication efficiency of JS5/05 in splenocytes was significantly higher than that of F48E8 and Herts/33, whereas replication efficiency of JS3/05 was partially inhibited by the early strong apoptosis (Figure 1). Thus, high replication capacity of genotype VIId NDV in splenocytes may be virus-dependent. Our results also suggest that *in vitro* replication of NDV may be determined by various factors, including cell type, viral genes and virus-induced CPE. Moreover, there may be an active interplay between virus replication and virus-induced cell death. At the early stage of infection, JS5/05 induced low-level apoptosis that allowed high virus replication, whereas excessive apoptosis induced by JS3/05 inhibited virus replication. Reversely, at the late phase of infection, JS5/05-induced cell death may promote virus replication, which may result from the failure of dead lymphocytes to clear virus and active virus release from leaky necrotic cells.

Our data are consistent with some previous findings. Rue et al. have demonstrated that virulent NDV elicits a robust host response in the spleen represented by hyperinduction of many cytokines, suggesting a correlation between pathogenicity and host innate immune response [10]. Ecco et al. have shown that the genotype VIId NDV strain ZJ1 is a stronger inducer of cytokine response than other virulent strains and the high-cytokine phenotype is consistent with viral pathogenicity and disease outcome [4].

Although there are many studies performed to elucidate virus pathogenicity and host response to NDV strains of varying virulence, evidence focusing on differences in virus-host interplay of different virulent isolates is limited. Our study presents *in vitro* evidence that differential viral modulation of host response corresponds with the contrasting histopathology phenotypes. Based on these results, we are performing animal experiments to provide supporting *in vivo* results to confirm this correlation.

## Conclusions

In summary, genotype VIId NDV isolates (JS5/05 and JS3/05) induced stronger innate immune and cell death responses than genotype IX NDV strain F48E8 and genotype IV NDV strain Herts/33 in chicken splenocytes. This *in vitro* evidence indicates that dysregulation of host response may contribute to severe tissue damage in the lymphoid system caused by genotype VIId NDV. This study also highlights that besides standard pathogenicity indices, evaluation of viral-host interaction is required for full characterization of NDV isolates.

## Methods

### Ethics statement

Experimental research on animals was approved by the Jiangsu Administrative Committee for Laboratory Animals (Permission number: SYXK-SU-2007-0005), and complied with the guidelines of Jiangsu laboratory animal welfare and ethical of Jiangsu Administrative Committee of Laboratory Animals.

### Viruses and cells

Details for viruses used in this study were listed in Table 1. Viruses were plaque-purified three times in CEF. These viruses were propagated in the allantoic cavities of 9-day-old specific-pathogen-free (SPF) embryonated chicken eggs. Virus titers were determined as 50% tissue culture infective dose (TCID_50_) in CEF.

Chicken splenocytes were isolated as previously reported [10]. Briefly, single-cell suspensions were prepared by cutting the spleens into small pieces, gently dissociating the spleen pieces on a sterile copper wire mesh, and then purified by gradient centrifugation (500 × g, 30 min) using Histopaque 1077 (Sigma, St. Louis, USA). After washing with phosphate-buffered saline (PBS) for three times, splenocytes were resuspended in RPMI 1640 medium (Invitrogen, CA, USA).

### *In vitro* infection

Viable cells were counted using trypan blue exclusion prior to infection. Total amount of splenocytes needed were inoculated with NDV strains at a multiplicity of infection (MOI) of 1. Infected cells were seeded at 1 × 10^6^ cells/well in 24-well-plates. Samples were collected at 6, 12 and 24 h pi.

### Virus replication assays

Viral replication was analyzed through two different ways. Virus contents in culture supernatants were quantified using standard plaque assay in CEF. In addition, transcription levels of viral M gene were determined using qRT-PCR. Primers for M genes (Table 2) were designed based on the conserved sequences that were identified using the Megalign program (DNASTAR, Madison, WI, USA).

**Table 2:** Primers for real-time PCR

**Target RNA**	**Primer sequence (5’-3’)**	**Accession no.**	**Product size (bp)**
IFN-α	Forward primer: CCACGACATCCTTCAGCACCT Reverse primer: TGAGGAGGCTTTGGCGTTG	NM_205427.1	89
IFN-β	Forward primer: TGCACAGCATCCTACTGCTCTTG Reverse primer: GTTGGCATCCTGGTGACGAA	NM_001024836.1	83
IFN-γ	Forward primer: AGCATTTGAACTGAGCCATCACC Reverse primer: CGTCAGCTACATCTGAATGACTTG	NM_205149.1	181
β-actin	Forward primer: ATTGTCCACCGCAAATGCTTC Reverse primer: AAATAAAGCCATGCCAATCTCGTC	NM_205518.1	113
M	Forward primer: GCTTGTGAAGGCGAGAGGTG Reverse primer: AACCTGGGGAGAGGCATTTG	-- ^a^	99

^a^: primers for viral M gene were designed based on the results of sequence comparison as described in the Methods.

### Cytokine gene expression

To determine host innate immune response to virus infection, the mRNA expression levels of IFN-α, IFN-β and IFN-γ were analyzed using a two-step qRT-PCR. Primers for these cytokine genes were listed in Table 2. Total RNA was isolated from splenocytes using the TRIzol reagent (Invitrogen, CA, USA). 100 nanogram (ng) of total RNA per sample was treated with 1 U DNase I (Fermentas, Maryland, USA) and used for reverse transcription reaction using 40 U M-MuLV reverse transcriptase (Fermentas, Maryland, USA) and 20 μM random primers in the presence of RNase inhibitor (Takara, Shiga, Japan) at 42°C for 90 min. cDNA and 200 nM (final concentration) each primer were mixed with 10 μl of 2 × SYBR Green PCR Master Mix (Takara). Reactions were performed in triplicate using the ABI Prism 7300 system with the following cycle profile: 1 cycle at 50°C for 2 min and 1 cycle at 95°C for 5 s followed by 40 cycles at 95°C for 5 s and 60°C for 31 s. 1 cycle for dissociation curve for all reactions was added. Relative expression levels were normalized using an internal β-actin control. The standard curve method was used to analyze the fold change of relative gene expression levels.

### Apoptosis assay

To measure virus-induced apoptosis, free nucleosomal DNA in cytoplasm was detected using the Cell Death Detection ELISA kit (Roche, Mannheim, Germany) according to the manual. Infected cells were pelleted by centrifugation and lysed with the lysis buffer from the kit. 20 μl of the cytoplasmic fraction of cell lysates was used to examine the release of free nucleosomal DNA.

### Annexin-V and PI double staining

NDV-induced CPE is associated with apoptosis. To evaluate morphological changes in infected cells, we used Annexin-V and PI that can differentiate changes in cellular structure in the process of programmed cell death. In the early stage of apoptosis, phosphatidylserine (PS) exposes upon the outer leaflet of the cell membrane, which can be detected by Annexin-V with high affinity. In the late stage of apoptosis, leaky necrotic cells release DNA that can be stained by PI. Annexin-V-FLUOS staining kit (Roche, Mannheim, Germany) was used to label splenocytes collected at 24 h pi as recommended by the producer.

### Statistical analysis

Statistical significance of differences between experimental groups was determined using the Independent-Samples T test. Values of *P* < 0.05 were considered significant.

## Abbreviations

CEF: Chicken embryo fibroblasts; CPE: Cytopathic effect; ELISA: Enzyme linked immunosorbent assay; IFN: Interferon; IRFs: IFN regulatory factors; M: Matrix; MOI: Multiplicity of infection; NDV: Newcastle disease virus; Ng: Nanogram; PBS: Phosphate-buffered saline; Pi: Post-inoculation; PI: Proidium iodid; PKR: Protein kinase R; PS: Phosphatidylserine; qRT-PCR: Quantitative real-time polymerase chain reaction; SPF: Specific-pathogen-free; TCID_50_: 50% tissue culture infective dose; TRAIL: TNF-related apoptosis-inducing ligand.

## Competing interests

The authors declare that they have no competing interests.

## Authors’ contributions

ZH and XL conceived and designed the experiments. ZH, JH and JZ performed the experiments. SH, XWL and XW contributed to the design of the study and revision of the draft. All the authors have read and approved the final manuscript.

## Authors’ information

^1^Animal Infectious Disease Laboratory, ^2^Ministry of Education Key Lab for Avian Preventive Medicine, School of Veterinary Medicine, Yangzhou University, 12 East Wenhui Road, Yangzhou, Jiangsu Province, 225009, China.
